# Bacterial infiltration and detorque at the implant abutment morse taper interface after masticatory simulation

**DOI:** 10.1038/s41598-022-20915-z

**Published:** 2022-10-12

**Authors:** Ana Paula Granja Scarabel Nogueira Bella, Alessandra Sayuri Tuzita, Ivana Barbosa Suffredini, Alberto Noriyuki Kojima, Elcio Magdalena Giovani, Alfredo Mikail Melo Mesquita

**Affiliations:** 1 Prosthodontics Department, Dental School, Universidade Paulista– UNIP, São Paulo, 04026-002 Brazil; 2 Environmental Pathology Department, Biodiversity Research Center, Universidade Paulista – UNIP, São Paulo, 04026-002 Brazil; 3 Prosthodontics Department, Dental School, Universidade Estadual Paulista Júlio de Mesquita Filho– UNESP, São José dos Campos, 16015-050 Brazil; 4 Oral Diagnosis and Dental Clinics Department, Dental School, Universidade Paulista – UNIP, São Paulo, 04026-002 Brazil; 5 Department of Prosthodontics, Universidade Paulista – UNIP, Av. do Cursino, 1919 1º andar – sala 10, Jardim da Saúde, São Paulo (SP) 04133-100 Brazil

**Keywords:** Fixed prosthodontics, Dental implants, Peri-implantitis

## Abstract

This study evaluated the bacterial infiltration and the detorque of indexed and non-indexed abutments of Morse taper implants (MTI) after mechanical cycling (MC). 40 MTI were distributed into four groups: IIA (indexed implant abutments); NIIA (non-indexed implant abutments); IIAMC (indexed implant abutments submitted to MC); NIIAMC (non-indexed implant abutments submitted to MC), which were carried out under one million 5 Hz frequency and 3 Bar pressure. After mechanical cycling, all groups were immersed in a bacterial solution in Brain Heart Infusion Agar. After detorque, the bacteria infiltration was evaluated by counting the colony-forming units. For the bacterial infiltration, analysis was applied to the Kruskal–Wallis test (*p* = 0.0176) followed by Dunn’s test. For the detorque analysis, the two-way repeated-measures ANOVA was applied, followed by the Tukey’s test (*p* < 0.0001). Bacteria infiltration was highly observed in NIIA (p = 0.0027) and were absent in IIAMC and NIIAMC. The detorque values for IIA (19.96Ncm ± 0.19Ncm), NIIA (19.90Ncm ± 0.83Ncm), and NIIAMC (19.51Ncm ± 0,69Ncm) were similar and remained close to the initial value, while IIAMC (55.2Ncm ± 2.36Ncm) showed an extremely significant torque value increase (p < 0.0001). The mechanical cycling resulted in mechanical sealing of the implant-abutment interface, preventing bacterial infiltration in the indexed and non-indexed specimens, and increasing the detorque strength in the group of indexed abutments.

## Introduction

Oral rehabilitation with dental implants requires more than just osseointegration success. The peri-implant tissues should also be in harmony with the existing dentition to achieve better functional and aesthetic rehabilitation^1^.

Regardless of the prosthetic platform, the implant-abutment interface is located below the gingival margin, where microbial infiltrations might occur, which can lead to bone loss through the inflammatory process, thus compromising not only the peri-implant tissues but also the osseointegration process^1–4^.

The long-term aesthetic and functional success of a prosthetic restoration supported by osseointegrated implants are causally related to the precision of the implant abutments adjustment^5^.

The preload applied on the abutment screw is a key mechanical factor to be taken into account, as it directly interferes with the stability of these components, creating a compressive tension of the screw/prosthetic abutment, abutment/implant, and prosthetic abutment/implant thread interfaces. The applied torque magnitude is causally related to the applied preload force and is limited by the screw resistance and by bone-implant interface resistance^6^.

The internal conical connection, designed for the Morse taper implant, shows a cone shape at the implant-abutment interface, where there is great mechanical retention between the connectors, being characterized by high contact pressure and resistance to friction between the internal implant surface and abutment, aiming to minimize abutment loosening and to improve mechanical stability, decreasing peri-implant bone resorption^7–9^. In the Morse taper connection, most of the load transfer from the abutment to the implant occurs through the taper connection, and the bending stress distribution occurs in the abutment screw^10^.

Periimplantitis is an inflammatory process that affects the soft and hard tissues around the implants, triggered by the accumulation of biofilm found in the oral cavity, observed in 10–50% of implant loss cases after the first year of implant load^1–17^. Thus, the periodontal and peri-implant health maintenance is essential, because the exacerbated and uncontrolled presence of pathogenic microorganisms in these peri-implant tissues induces, over time, the osseointegration loss and consequently leads to implant loss^17,18^.

The purpose of this study was to evaluate the bacterial infiltration and the detorque values of indexed and non-indexed abutments of Morse taper implants after mechanical cycling.

This study’s null hypothesis is that mechanical cycling and indexing of abutments do not interfere with detorque nor with bacterial infiltration.

## Materials and methods

### Experimental design

Forty 3.5 × 10 mm Morse taper implants (MTI), manufactured and commercialized by SIN (São Paulo, Brazil, batch #QO60200316), were randomly divided into four groups of 10 implants each. One extra implant from each group was used to validate the bacterial contamination hypothesis (n = 10/per group, plus the extra one).

**Figure 1:**
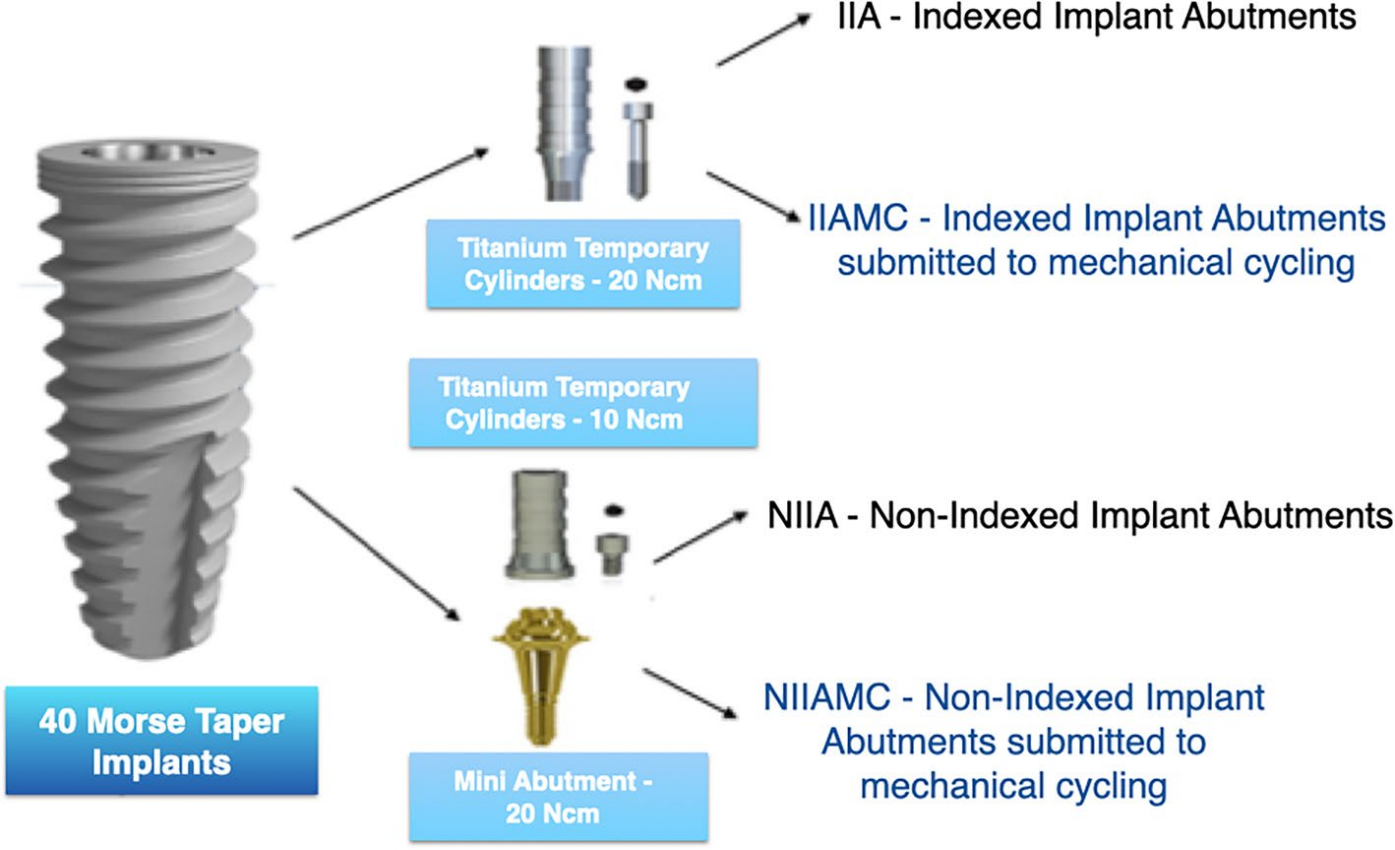
Representative scheme of the indexed Morse taper implant, with the respective indexed and non-indexed abutments, on which the 40 sets of implants and prosthetic abutments for groups IIA, NIIA, IIAMC and NIIAMC were mounted.

As shown in Fig. 1, as determined by the sample calculation of the pilot study performed before the experiments analyzed in this study, indexed titanium temporary cylinders were installed (with a torque of 20 Ncm) in the specimens of groups IIA and IIAMC. In groups NIIA and NIIAMC, abutments were installed on the non-indexed mini abutments (with a torque of 20Ncm), and on these, titanium temporary cylinders (with a torque of 10Ncm) were installed. Groups IIAMC and NIIAMC underwent mechanical cycling.

### Assembling of implant sets

**Figure 2:**
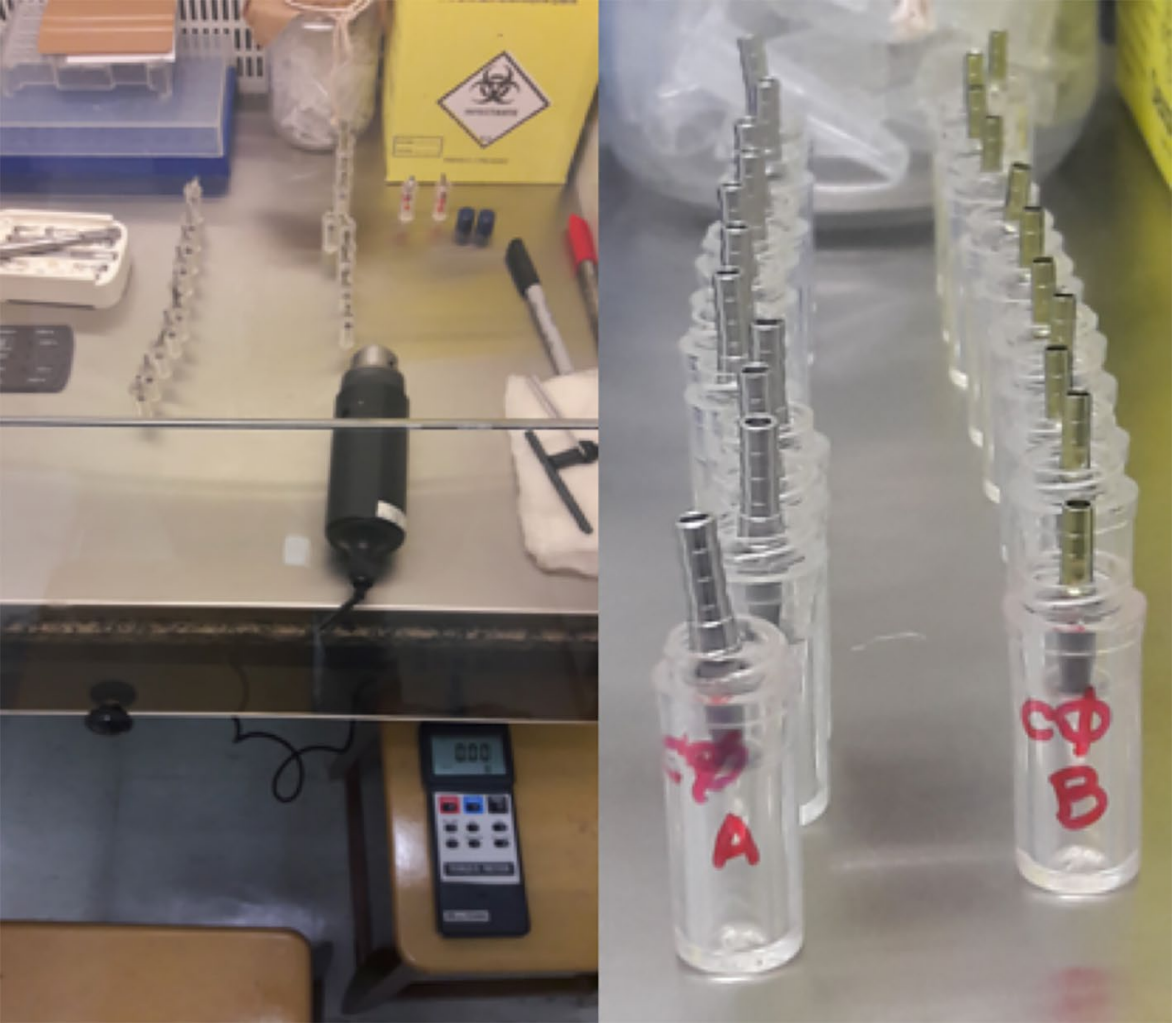
Sets of implants and abutments mounted in a laminar flow with aid of a digital torque wrench.

The assembling of implant sets for each group was performed in a laminar flow with the aid of a digital torque wrench, following the manufacturer’s recommendation (Fig. 2).

IIA and NIIA non-cycled were contaminated to assess a possible bacterial infiltration. IIAMC and NIIAMC underwent mechanical cycling then, together with IIA and NIIA, underwent bacterial contamination and the abutments were removed for the evaluation of the detorque values and the internal implant contamination.

### Mechanical cycling

Mechanical cycling is a method to simulate masticatory cycles and to replicate and evaluate the behavior of implant systems, the standard ISO 14801:2007 was developed to standardize these tests using cyclic fatigue^19^.

After connecting all implants to their respective abutments, the specimens from groups NIIA and NIIAMC were attached to an acrylic resin die, in a 1/2 PVC pipe jointer, with the aid of a metal clamp to secure the axial position of the sets to be submitted to mechanical cycle loads, in an automatic machine (Biocycle—BIOPDI, São Carlos, Brazil), where continuous cycles simulated the masticatory load. A 75 cycle/per minute load was applied over the implant sets, which mimicked the human masticatory frequency^6^.

**Figure 3:**
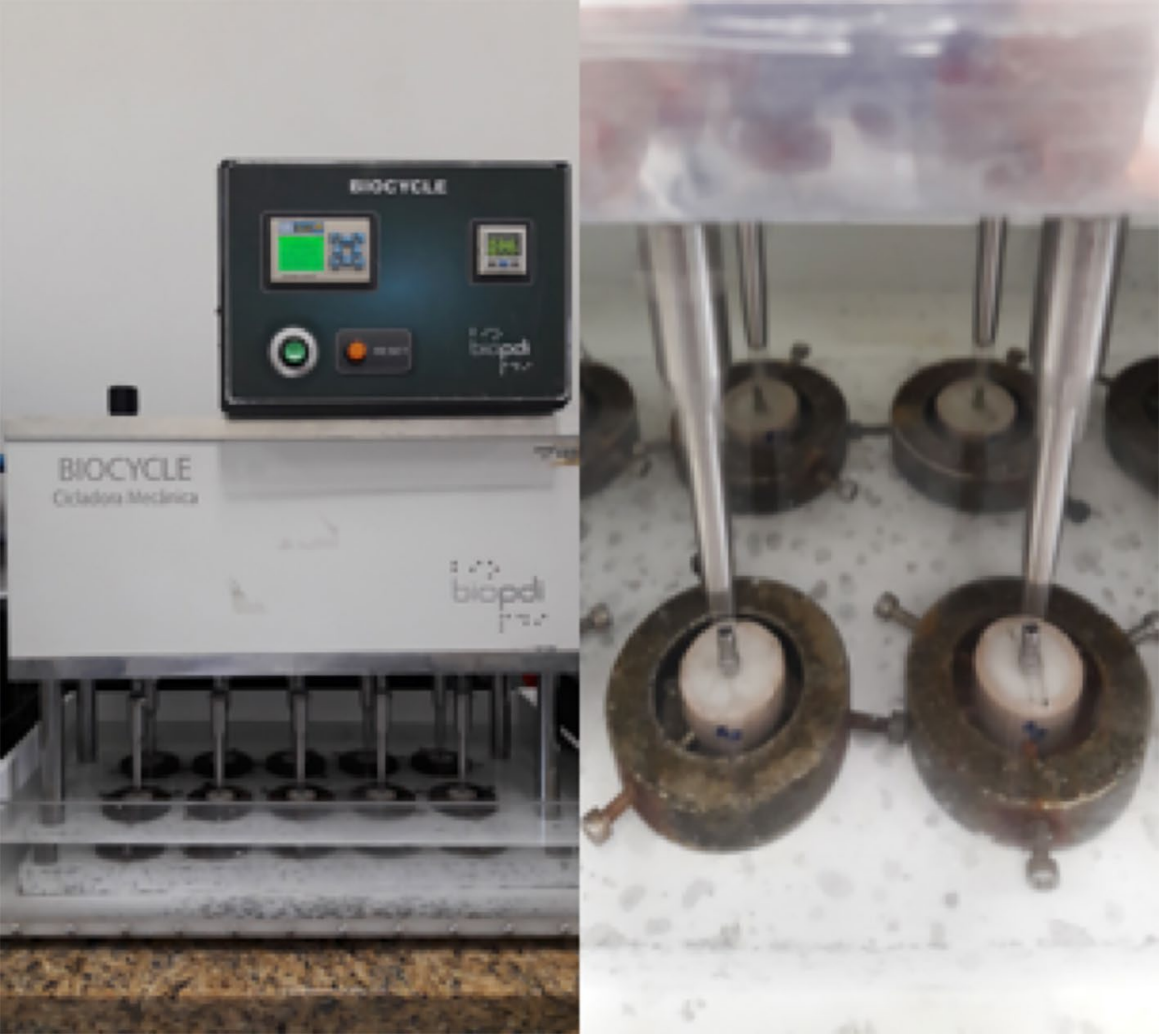
Representation of mechanical cycling in IIAMC.

The mechanical cycling tests (1.000.000 cycles) were performed with the implant sets immersed in distilled water at 37 ºC, under a frequency of 5 Hz and 3 Bar pressure (0.306 MPa, ~ / ± 150 Ncm force/load) (15.02 kgf, equivalent to 147.26 Ncm load), using 25 mm diameter pistons with maximum extension. The mechanical tests were performed under the standardized protocol for implant fatigue tests, developed by the International Organization for Standardization in 2003—ISO 14801, designed for single implants, tested under highly demanding clinical setups, which allowed minimum mechanical behavioral conditions and comparisons between the tested systems to be applied on the same test protocol (Fig. 3).

After the mechanical cycling process of groups NIIA and NIIAMC, the acrylic resin die sets were removed using a low-rotation straight handpiece and a tungsten bur sterilized in an autoclave. These groups underwent the same process of bacterial infiltration test applied to groups IIA and IIAMC.

### Bacterial contamination

#### Preparation of the bacteria strain

*Streptococcus mutans* (*Smut*) (ATCC™ 25,175®) was cultivated in Brain Heart Infusion Agar (BHIA), in an incubator at 36° ± 2 °C for 48 h, in Petri dishes. From the primary culture, a 24 h freshly culture was obtained in BHIA, from which 400 mL of bacterial suspension were prepared at the final concentration of 3.48 × 10^8^ CFU/mL. The suspension was applied to perform the bacterial infiltration technique.

### Description of the bacterial infiltration evaluation technique

**Figure 4:**
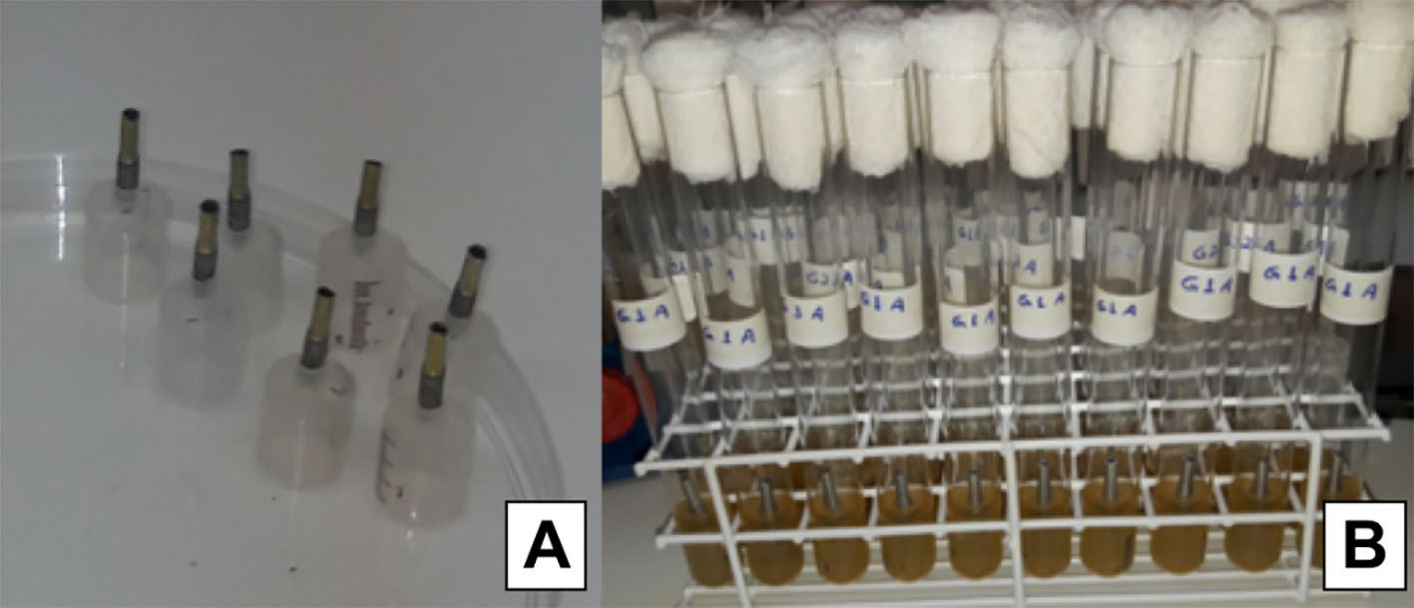
(**A**) Sets of implants and abutments mounted to the syringes; (**B**) Syringes with the implant-abutment specimens inserted with bacterial culture medium in the test tubes.

For the evaluation of bacterial infiltration, an innovative and low-cost technique, which is described elsewhere, was used^20^. Disposable 5 mL syringes were prepared as stands to hold the 44 specimens (n = 10 per group; one set from each group was used as negative control), which were cut on the 3 mL mark height, leaving them with a length of 42.35 mm. The needle-fitting nozzles were also sawn and widened up to a diameter to fit the implants used up to their third middle section, that is, 2.35 mm. In addition, two holes with a diameter of 3.95 mm were made, next to the nozzle area, on the syringes’ upper portion. After that, the syringes were assembled, in laminar flow, with the cycled and not cycled implant-abutment specimens (Fig. 4A). With such dimensions, the syringes could be inserted into the test tubes measuring 16 cm in length × 1.42 cm internal diameter and 1.46 mm external diameter (Fig. 4B).

**Figure 5:**
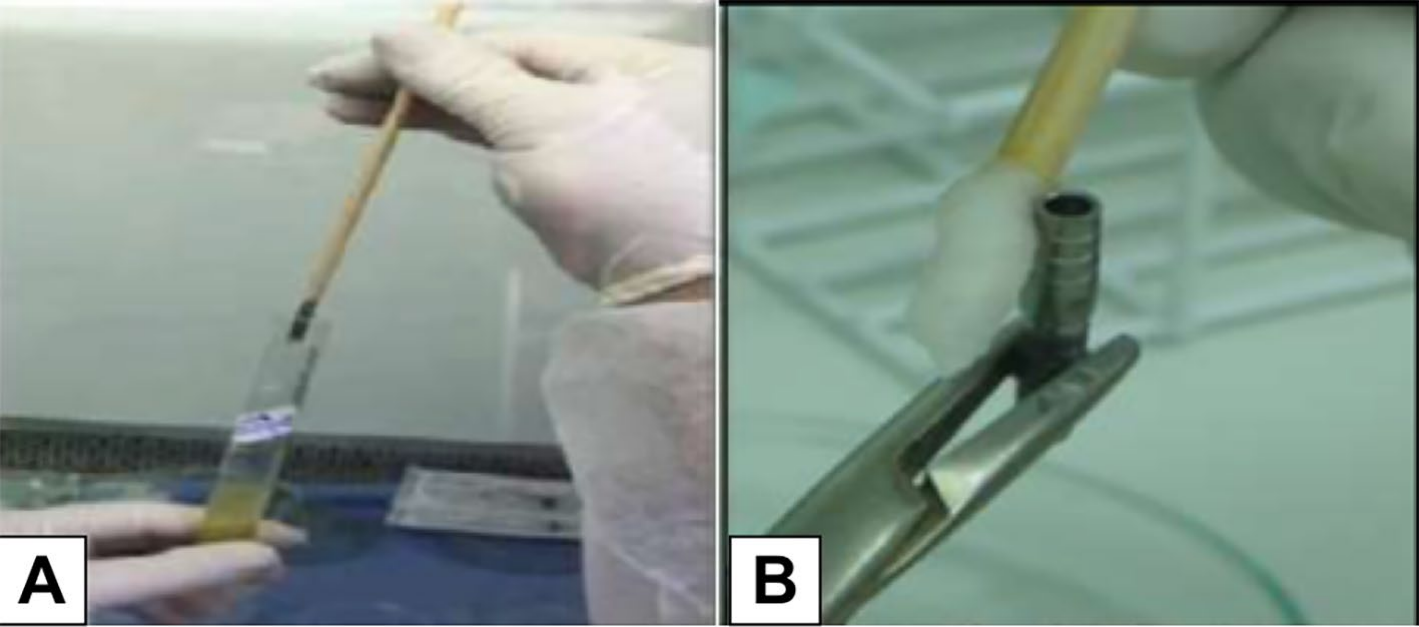
(**A**) The removal of the syringe from the test tube using a thin swab; (**B**) Cleaning of the external surface of the implant-abutment set with a chlorhexidine solution at 0.12%.

A total of 9.5 mL of the prepared bacterial suspension were transferred to each of the test tubes, which were covered with hydrophobic gauze pads. A test tube with innocuous BHIA was used as a negative control. The test tubes were taken into the incubator at 36° ± 2 °C for 48 h. After that period, each implant was removed with the assistance of a sterile thin wood stick, and inserted into the hole made in the upper part of the syringe (Fig. 5A). The implant was removed from the syringe support and was immediately cleaned with a chlorhexidine solution at 0.12%, using a flexible swab (Fig. 5B).

#### Detorque and bacterial count

**Figure 6:**
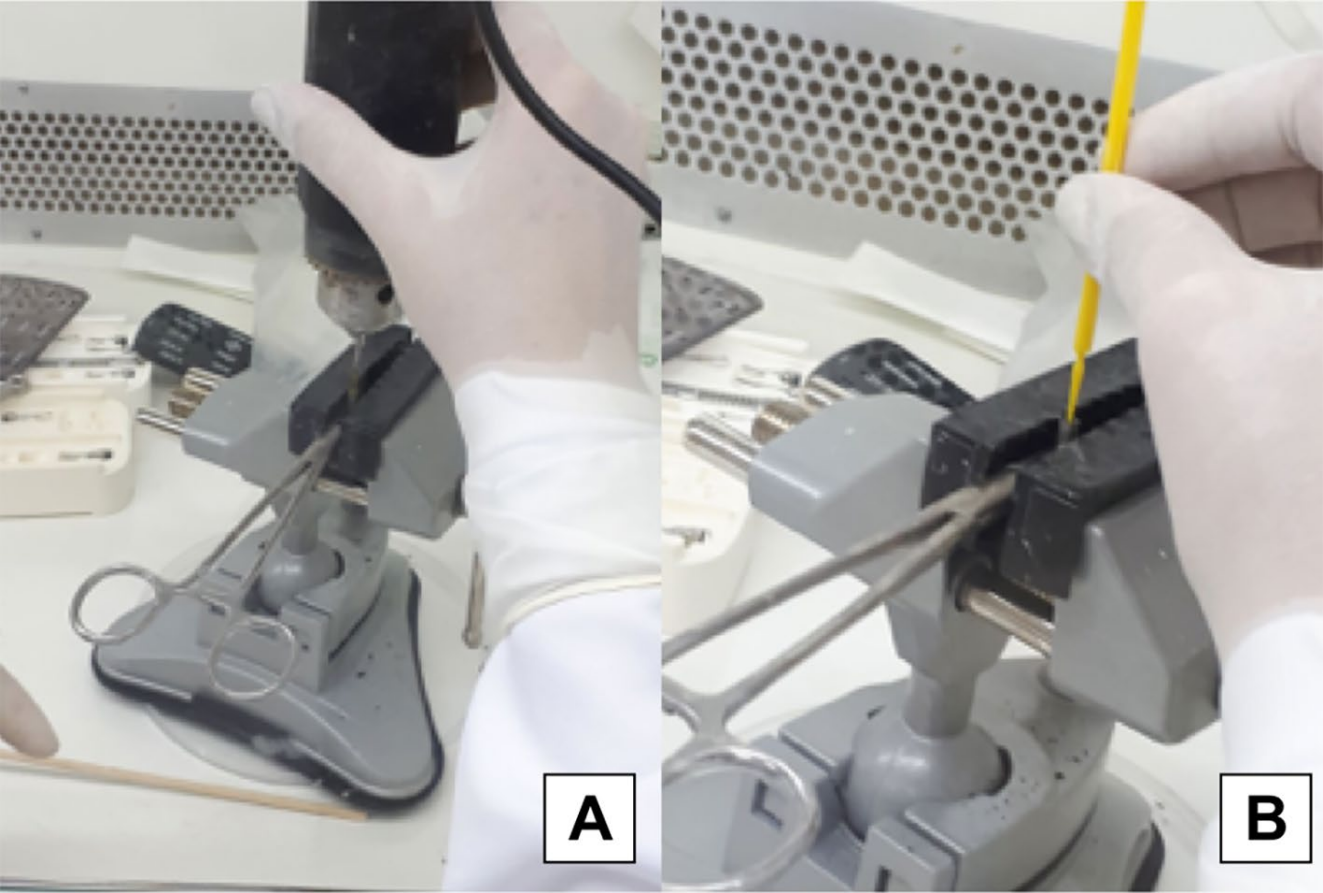
(**A**) Detorque of the abutment-implant set with the digital torque wrench; (**B**) Sample collection from the inner side of the implant after detorque.

**Figure 7:**
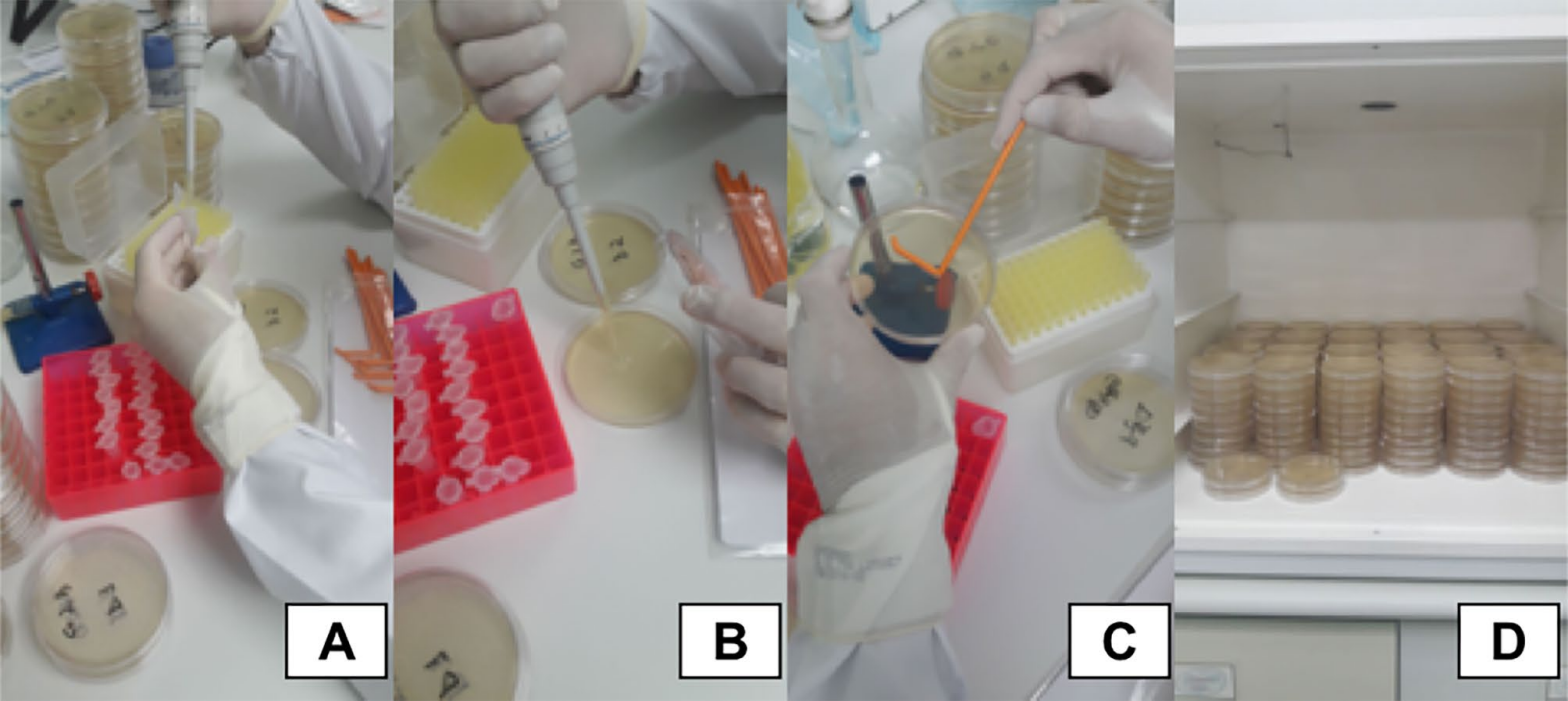
(**A**) Eppendorf tubes containing saline solution; (**B**) Bacterial solution transferred to a Petri dish with BHIA; (**C**) Harvesting of bacterial subculture in a Petri dish; (**D**) Subculture dishes in a bacterial growth chamber.

All implant sets were subjected to detorque using a digital torque wrench, to evaluate the torque resistance (Fig. 6A). After that, bacteria that have infiltrated into the internal part of the implants were collected with a sterile swab (Fig. 6B) and immediately transferred to a 1.5 mL Eppendorf vial containing 1 mL of saline solution (Fig. 7A).

The tubes were homogenized in a vortex for 30 s, and then, a 1/10 rate dilution was made in a second vial, by adding 100 µL to 900 µL of saline solution. Then, the tubes were homogenized again. Subcultures for each of the dilutions were made in Petri dishes containing BHIA (Fig. 7B), so that each dish received 100 µL of the respective suspension, in duplicate (Fig. 7C). Subcultures were incubated at 35° ± 2 °C for 48 h (Fig. 7D). The colony-forming unit (CFU/mL) count was performed to define the bacterial load that infiltrated each specimen.

### Statistical analysis

All data were submitted to a normality assay based on the Shapiro–Wilk test. For detorque analysis (normality for IIA = 0.8657; NIIA = 0.0256; IIAMC = 0.0545; NIIAMC = 0.0680), two-way repeated-measures ANOVA was performed, followed by the Tukey’s test. For the analysis of bacterial infiltration (normality for IIA = p < 0.0001; NIIA = p = 0.0002; IIAMC = p < 0.0001; NIIAMC = p < 0.0001), the Kruskal–Wallis test was performed, followed by Dunn’s statistical test. For all tests, a significance of *α* < 0.05 was considered (GraphPad Prism 7.0).

## Results

### Detorque

**Figure 8:**
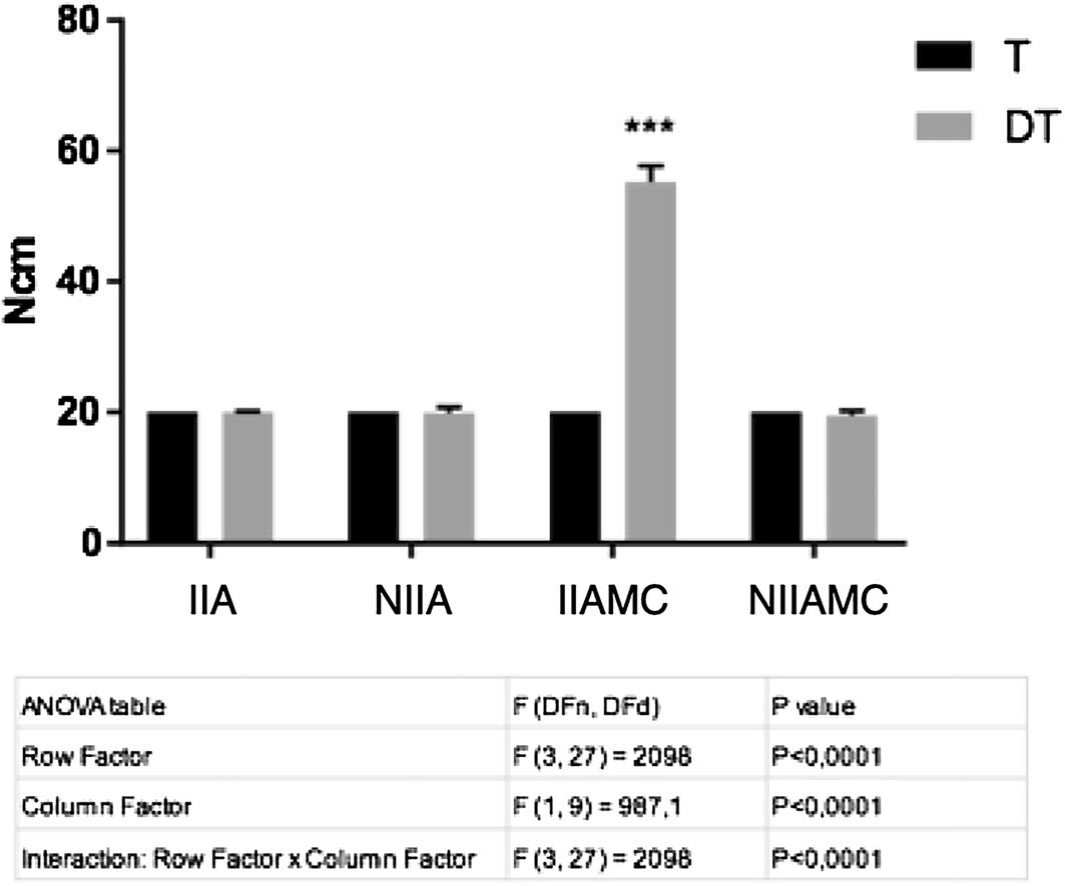
Statistical analysis of the detorque values of the specimens—Two-Way Repeated Measures ANOVA, followed by Tukey’s test.

The detorque evaluation was made by considering two variables: torque/detorque and the different types of prosthetic abutments. Significance was observed in the torque variable (F_(3,27)_ = 2098; *p* < 0.0001), in the type of prosthetic abutment (F_(1,9)_ = 987.1; *p* < 0.0001) and in the interaction between the two variables (F_(3,27)_ = 2098; *p* < 0.0001) (Fig. 8). According to the statistical results, the following detorque mean and standard deviation values were measured: IIA = 19.96Ncm ± 0.19Ncm; NIIA = 19.9Ncm ± 0.83Ncm; IIAMC = 55.2Ncm ± 2.36Ncm and NIIAMC = 19.51Ncm ± 0.69Ncm. No differences were observed between IIA, NIIA, and NIIAMC (p > 0.05), while IIAMC showed an expressive significant improvement in the detorque value (*p* < 0.0001), regarding the initial torque of 20 Ncm.

### Bacterial infiltration

**Figure 9:**
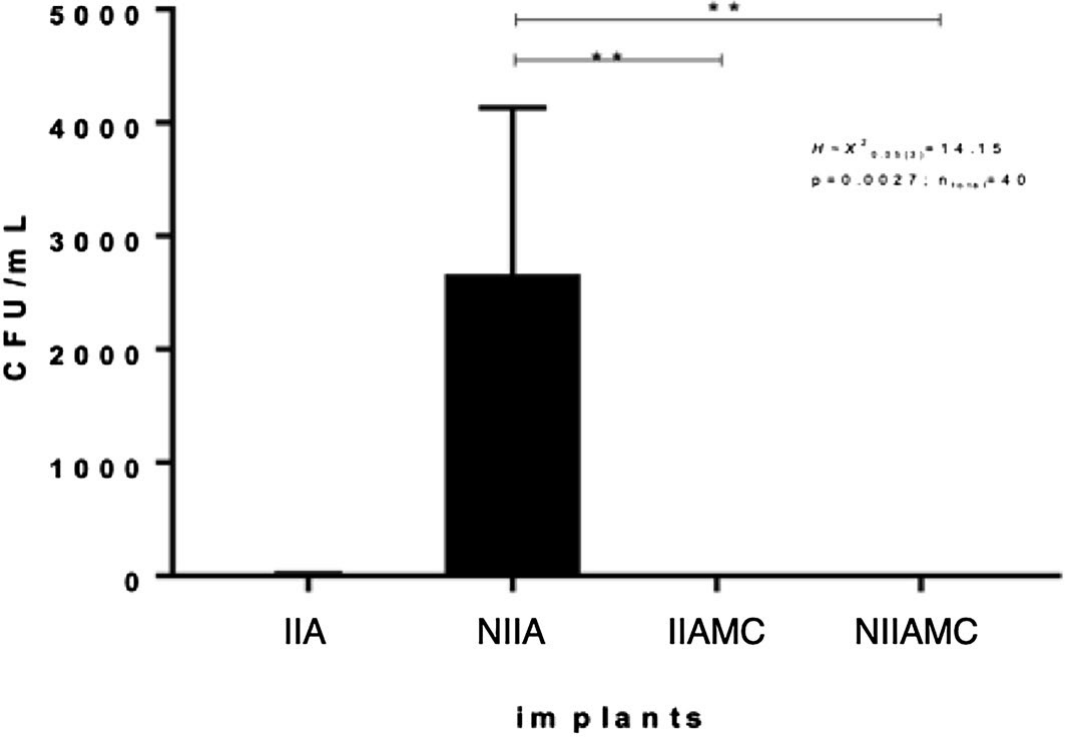
Non-parametric analysis—Kruskal–Wallis and Dunn’s tests (*α* < 0,05): *IIA* indexed abutments and Morse taper implants, *NIIA* non-indexed abutments and Morse taper implants; *IIAMC* mechanical cycling indexed abutments and Morse taper implants, *NIIAMC* Mechanical cycling non-indexed abutments and Morse taper implants.

According to the statistical results, bacteria have massively infiltrated in NIIA (H ~ _%_^2^_0.05(3)_ = 14.15; *p* = 0.0027; n_total_ = 40), in relation to IIAMC (p = 0.0076) and to NIIAMC (p = 0.0076) (Fig. 9).

## Discussion

The null hypothesis was rejected in this study, as mechanical cycling altered the behavior of the indexed and non-indexed specimens, both for bacterial infiltration and for detorque values.

Studies comparing implant prosthetic connections show that the lack of adaptation between implants and abutment can result in higher tension around the implant, bone, and implants connections, which can be aggravated by mechanical cycling, leading to a decrease in detorque values^11–14^, which can cause screw loosening, as demonstrated by some prospective clinical studies with 3 to 5 years of follow-up^15,16^.

Although there are many studies on bacterial infiltration, the literature is still scarce of articles that compare indexed and non-indexed abutments with the performance of mechanical cycling. According to the results obtained in this study, it was shown that there is no difference in bacterial infiltration between the two types of abutments, but there are statistically significant differences when subjected to mechanical cycling, highlighting its importance.

Chandra et al.^21^ simulated the masticatory load of a 6-month in-function prosthesis, correlating it to 500.000 mechanical cycles. However, for other authors, 100.000 mechanical cycles correspond to five years of chewing^10,22^. The methodology of this study was performed in such a way that the mechanical cycles and loads simulated 4.5 years of masticatory function, replicated, and evaluated the performance of implant systems, using the ISO 14,801:2007 standard^3,5,19,23^.

After 1,000,000 cycles of the mechanical cycling tests, which occurred without the fracture of the implant abutments screws, the group NIIAMC maintained values close to the initial torque value when the abutment-implant sets were submitted to detorque. In contrast, the group IIAMC with a Morse taper implant and an indexed abutment subjected to mechanical cycling achieved a mean detorque value of 55.20 Ncm compared to the initial torque value of 20 Ncm and was significant to the other groups, demonstrating the clinical relevance of achieving greater mechanical interlock between the Morse taper implant / prosthetic abutment connection after simulating masticatory loading of approximately 4.5 years.

For the group NIIAMC, the influence of the mechanical cycling was significant because it directly interferes with the sealing capacity of the implant-abutment interface, which made bacterial infiltration impossible.

In this study, 1 million cycles were performed, with no loosening of the implant-abutments screws being identified. The detorque values of IIA, NIIA, and NIIAMC were similar, however, for IIAMC, the values were higher.

The indexed Morse taper connection presents an internal conical design that promotes an intimate adaptation between the surfaces during the abutment installation into the implant, reaching a mechanical resistance similar to that of a single-piece implant, and improving the mechanical properties and the stability of the abutment, preventing its loosening and maintaining high resistance to the opening force, due to the maintenance of the friction coefficient between the components, thus ensuring excellent prosthetic stability, as reported in previous studies^3,9,10,24–27^.

Recent studies have shown that the Morse taper implant connection has a better sealing capacity concerning the space between implant-abutment to the loss of the bone crest^6,9,10,28,29^. The Morse taper implant appears to be more efficient regarding biological aspects, favoring less bacterial infiltration and bone loss in single implants, including aesthetic dental areas. It can also be successfully indicated for fixed partial dentures and overdentures^30^.

Tuzzolo et al.^31^ performed a comparative analysis of the mechanical resistance of implants of different diameters, showing that the implants of smaller diameter provided less fracture resistance, both in the tensile strength tests and in the maximum bending moment, mainly about the single-piece extra-narrow and single-piece narrow implants that deformed in the implant body area, thus demonstrating that the implant diameter selected and the use of intermediate abutments is also a factor of the utmost clinical importance.

The deformation resistance and fracture on the oblique forces loads prove that the Morse taper implant connection presents significantly better results, due to the solid design of the prosthetic abutment and the friction locking mechanism^9^.

In this study, the mechanical cycling process influenced the prevention of bacterial infiltration, as observed in the mechanically activated indexed and non-indexed groups (IIAMC and NIIAMC) and had greater statistical significance for the non-indexed and non-cycled group (NIIA) with a higher rate of bacterial infiltration.

The infiltration of saliva through the implant-abutment interface in different types of implant connections (external hexagon, internal hexagon, and morse taper implant), with and without load, was confirmed in some studies and found microorganisms present on the internal surface of all groups, however, the groups with the Morse taper connection showed results with lower infiltration values, both when submitted and not submitted to load^5,28^.

The implant’s sealing capacity to prevent bacterial infiltration is granted by the mechanical sealing and locking provided by macro geometry and internal conical connection, which is hermetic regarding bacterial infiltration, in vitro^32^. In the comparison of bacterial sealing between the implant and the abutment both solid and pass-through-screw types, no statistical difference was found, neither concerning the number of bacteria colonies nor the percentage of bacterial infiltration^33^. Implant-abutment interface contamination has been studied as to its influence on the inflammation of the adjacent tissues, which can manifest itself through mucositis and/or peri-implantitis, resulting in the loss of the dental implant^1,34,35^.

To evaluate the bacterial infiltration in this study, a new testing technique was proposed from the implants’ external to the internal environment, representing a more accurate and financially viable situation in the clinical routine. Various techniques for the verification of implant contaminations and bacterial infiltration have been recommended in several studies^30,36,37^.

*Smut*, in BHIA, was used for being an important microorganism that occurs in the oral cavity as its natural habitat, in healthy and good oral hygiene conditions. Calan et al.^38^ described the presence of implant-abutment interface contamination for one or more periodontal odontogenic microorganisms that inhabit the internal part of the implant, even though there is no difference between the colonization of an individual species of microorganisms when compared to the different regions of implant placement, in vivo.

The presence and quantity of oral lubricant (saliva, peri-implant fluid, and/or blood) between the implant components can affect the friction coefficient between the Morse taper connection components, decreasing this coefficient as the number of lubricants increases^24^.

Initially, the presence or absence of index was not relevant in the bacterial infiltration evaluation results, as both groups presented bacterial infiltration. After mechanical cycling simulating chewing, groups IIAMC and NIIAMC demonstrated an absence of bacteria in the bacterial infiltration test.

However, regarding the bacterial infiltration, the hermetic sealing of the specimens and cemented abutments provide low bacterial permeability in the conical types connections and a high incidence of infiltration in screwed connections^25^.

An important and quite relevant factor for the evaluation of the stability of the implant-abutment connections is the detorque values, as per the preload value remaining in the screw, after mechanical cycling^12,39^. The crucial reason for the loss of the implant prosthetic abutment, in an external hexagon or internal hexagon connection, is the loss of the abutment screw preload and the resulting unscrewing or fatigue failure of the screw material^39^.

Detorque values close to or higher than the initial torque values indicate a good prognosis for the connections in question^5^. Mechanical cycling promotes movements that simulate human chewing movements and mimics the distortion and fatigue of the implant-abutment specimens^21^.

The detorque values of prosthetic abutments applied in the Morse cone system tend to decrease as the number of cycles of insertion and removal of the abutment increases^22^.

To improve the knowledge regarding the contamination process that can occur in the union space between the implant-abutment, in indexed Morse taper implants, with indexed and non-indexed abutments, this study was performed using the analysis technique of bacterial infiltration, described in the methodology, searching for greater approximation with the reality of the oral environment.

Although these results were obtained, in another in vitro study, with the same purpose, however, with contamination from the internal environment to the external environment of the implant, the mechanical behavior and the bacterial micro-infiltration in the implant-abutment conical interface, after mechanical cycling, showed no statistically significant difference between the removal force, reverse torque and contamination values when compared to implants of the same type. The abutment removal force or detorque was not affected by mechanical cycling, since the bacterial sealing of the implant-abutment conical interface was not effective, in any of the analyzed conditions. Inaccurate mechanical activation of implants and abutments does not allow a surface area that provides effective frictional sealing and bacterial infiltration. In clinical relevance, the microscopic space caused by the desadaptation of the implant-abutment, superficial irregularities, and plastic deformation of all parts allow bacterial contamination of oral implants^24^.

The precise application of the technique used in this study, after mechanical cycling, within the parameters historically analyzed and validated in the literature, resulted in the finding of significant results in the investigation of the proposed hypothesis. The mechanical activation process favors the mechanical overlap of the implants and abutment specimens regarding the bacterial infiltration of the indexed and non-indexed Morse taper implants, biostatistically significant for the non-indexed prosthetic abutments, becoming of the utmost importance, as well as literature states that the Morse taper connections tend to have intimate contact between surfaces. Besides that, the initial torque recommended by the manufacturer must be maintained, because it was noted that the initial torque remained close to the detorque value of the specimens with indexed and non-indexed non-cycled abutments, and it was only higher for the sets with cycled indexed abutments. Bacterial infiltration, in vitro, was only observed in Morse taper implants with non-indexed and non-cycled prosthetic abutments.

As a limitation of this study, only a single bacterial strain was used, stating that the data at hand suggest that these are just preliminary tests, so in the future, further in vitro studies should be conducted to test other bacterial strains present in the oral cavity, including pathogenic bacteria in periodontitis or peri-implantitis, using this new bacterial infiltration technique to test the ability of the infiltration range between different bacteria.

From the prosthetic rehabilitation perspective, the use of the indexed components can facilitate prosthesis manufacturing, because there is the possibility of transferring the implant platform to the working cast, which can help choose the best prosthetic abutment. However, the clinical disadvantage is to possibly reduce the implant’s frictional retention area, due to the presence of the index, influencing the bacterial infiltration and the detorque of the abutment screws.

## Conclusion

According to the results obtained and the in vitro study limitations, it may be concluded that:

1. The presence of the index increased the detorque resistance of the prosthetic abutments in face of mechanical cycling.
2. There was no bacterial infiltration regardless of the presence of the index after mechanical cycling.

## Supplementary Information

Supplementary Information 1.

Supplementary Information 2.

## Data Availability

All data generated or analysed during this study are included in this published article (and its Supplementary Information files).
