# Supplementary material for: Bacterial infiltration and detorque at the implant abutment morse taper interface after masticatory simulation
Source: Sci Rep. 2022 Oct 12;12:17103. doi: 10.1038/s41598-022-20915-z (PMC9556662; doi:10.1038/s41598-022-20915-z)

# **RAW DATA**

**Table 1 –** Detorque measurement for implant sets and implant abutments, with the respective mean and standard deviation values.

|  | **IIA** | **NIIA** | **IIAMC** | **NIIAMC** |
| --- | --- | --- | --- | --- |
| **Sample 1** | 19,60 | 18,00 | 55,00 | 17,80 |
| **Sample 2** | 20,00 | 20,00 | 56,00 | 19,70 |
| **Sample 3** | 19,80 | 20,00 | 58,00 | 19,90 |
| **Sample 4** | 20,00 | 21,00 | 55,00 | 20,30 |
| **Sample 5** | 20,10 | 21,00 | 58,00 | 20,10 |
| **Sample 6** | 20,00 | 19,00 | 52,00 | 18,90 |
| **Sample 7** | 20,30 | 20,00 | 58,00 | 19,70 |
| **Sample 8** | 19,80 | 20,00 | 52,00 | 19,20 |
| **Sample 9** | 19,90 | 20,00 | 56,00 | 19,90 |
| **Sample 10** | 20,10 | 20,00 | 52,00 | 19,60 |
| **Mean value** | 19,96 | 19,90 | 55,20 | 19,51 |
| **Standard Deviation Value** | 0,19 | 0,83 | 2,36 | 0,69 |

**IIA –** Indexed Morse taper implants and indexed implant abutments;

**NIIA –** Indexed Morse taper implants and non-indexed implant abutments;

**IIAMC –** Indexed Morse taper implants and indexed implant abutments submitted to mechanical cycling;

**NIIAMC –** Indexed Morse taper implants and non-indexed implant abutments submitted to mechanical cycling.

**Table 2 –** Results of bacterial colony forming units counts in the first dilution of the bacterial suspension of *Streptococcus mutans*, in Brain Heart Infusion Agar, after bacterial infiltration technique in the implant sets and implant abutments.

| **Dilution D1** |  |  |  |  |
| --- | --- | --- | --- | --- |
|  | **IIA** | **NIIA** | **IIAMC** | **NIIAMC** |
| **Sample 1** | 0 | 10 | 0 | 0 |
| **Sample 2** | 0 | 12354 | 0 | 0.0001 |
| **Sample 3** | 0 | 10 | 0 | 0 |
| **Sample 4** | 0 | 185 | 0 | 0 |
| **Sample 5** | 0 | 0 | 0.0001 | 0 |
| **Sample 6** | 0 | 4480 | 0 | 0 |
| **Sample 7** | 0 | 9595 | 0 | 0 |
| **Sample 8** | 0 | 5 | 0 | 0 |
| **Sample 9** | 60 | 0 | 0 | 0 |
| **Sample 10** | 60 | 0 | 0 | 0 |

**IIA –** Indexed Morse taper implants and indexed implant abutments;

**NIIA –** Indexed Morse taper implants and non-indexed implant abutments;

**IIAMC –** Indexed Morse taper implants and indexed implant abutments submitted to mechanical cycling;

**NIIAMC –** Indexed Morse taper implants and non-indexed implant abutments submitted to mechanical cycling.

**Table 3 –** Analysis of the bacterial contamination, with reference to the first dilution (D1) of the bacterial suspension of *Streptococcus mutans* for all groups.


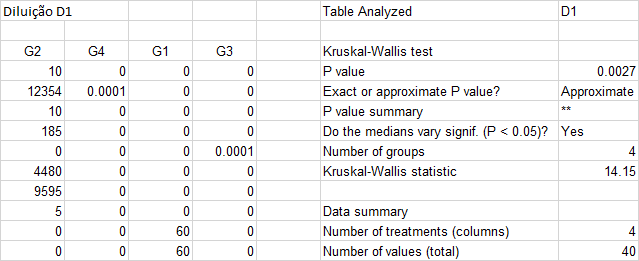

Supplement: Supplementary file 1 — Supplementary Information 1. [file 41598_2022_20915_MOESM1_ESM.docx]
