# Supplementary material for: Bacterial infiltration and detorque at the implant abutment morse taper interface after masticatory simulation
Source: Sci Rep. 2022 Oct 12;12:17103. doi: 10.1038/s41598-022-20915-z (PMC9556662; doi:10.1038/s41598-022-20915-z)
Supplement: Supplementary file 2 — Supplementary Information 2. [file 41598_2022_20915_MOESM2_ESM.docx]

**SUPPLEMMENTARY MATERIAL**

**Statistical Analysis**

**DATA TORQUE – DETORQUE – BACTRIAL INFILTRATION –**

**CRUDE DATA BACTERIAL INFILTRATION CFU/ML**

| G1 | G2 | G3 | G4 |
| --- | --- | --- | --- |
| 0 | 10 | 0 | 0 |
| 0 | 12354 | 0.0001 | 0 |
| 0 | 10 | 0 | 0 |
| 0 | 185 | 0 | 0 |
| 0 | 0 | 0 | 0.0001 |
| 0 | 4480 | 0 | 0 |
| 0 | 9595 | 0 | 0 |
| 0 | 5 | 0 | 0 |
| 60 | 0 | 0 | 0 |
| 60 | 0 | 0 | 0 |

**NORMALITY TEST BACTERIAL INFILTRATION**

|  | G1 | G2 | G3 | G4 |
| --- | --- | --- | --- | --- |
| Number of values | 10 | 10 | 10 | 10 |
|  |  |  |  |  |
| Minimum | 0 | 0 | 0 | 0 |
| 25% Percentile | 0 | 0 | 0 | 0 |
| Median | 0 | 10 | 0 | 0 |
| 75% Percentile | 15 | 5759 | 0 | 0 |
| Maximum | 60 | 12354 | 0.0001 | 0.0001 |
|  |  |  |  |  |
| Mean | 12 | 2664 | 1e-005 | 1e-005 |
| Std. Deviation | 25.3 | 4641 | 3.162e-005 | 3.162e-005 |
| Std. Error of Mean | 8 | 1468 | 1e-005 | 1e-005 |
|  |  |  |  |  |
| Lower 95% CI of mean | -6.097 | -655.9 | -1.262e-005 | -1.262e-005 |
| Upper 95% CI of mean | 30.1 | 5984 | 3.262e-005 | 3.262e-005 |
|  |  |  |  |  |
| Sum | 120 | 26639 | 0.0001 | 0.0001 |
|  |  |  |  |  |
| Shapiro-Wilk normality test |  |  |  |  |
| W | 0.5093 | 0.6502 | 0.3657 | 0.3657 |
| P value | <0.0001 | 0.0002 | <0.0001 | <0.0001 |
| Passed normality test (alpha=0.05)? | No | No | No | No |
| P value summary | **** | *** | **** | **** |
|  |  |  |  |  |
| KS normality test |  |  |  |  |
| KS distance | 0.4824 | 0.4034 | 0.5241 | 0.5241 |
| P value | <0.0001 | <0.0001 | <0.0001 | <0.0001 |
| Passed normality test (alpha=0.05)? | No | No | No | No |
| P value summary | **** | **** | **** | **** |

**KRUSKAL-WALLIS BACTERIAL INFILTRATION**

| Table Analyzed | D1 |
| --- | --- |
|  |  |
| Kruskal-Wallis test |  |
| P value | 0.0027 |
| Exact or approximate P value? | Approximate |
| P value summary | ** |
| Do the medians vary signif. (P < 0.05)? | Yes |
| Number of groups | 4 |
| Kruskal-Wallis statistic | 14.15 |
|  |  |
| Data summary |  |
| Number of treatments (columns) | 4 |
| Number of values (total) | 40 |

| Number of families | 1 |  |  |  |  |
| --- | --- | --- | --- | --- | --- |
| Number of comparisons per family | 6 |  |  |  |  |
| Alpha | 0.05 |  |  |  |  |
|  |  |  |  |  |  |
| Dunn's multiple comparisons test | Mean rank diff. | Significant? | Summary | Adjusted P Value |  |
|  |  |  |  |  |  |
| G1 vs. G2 | -10.7 | No | ns | 0.0557 | A-B |
| G1 vs. G3 | 2.55 | No | ns | >0.9999 | A-C |
| G1 vs. G4 | 2.55 | No | ns | >0.9999 | A-D |
| G2 vs. G3 | 13.25 | Yes | ** | 0.0076 | B-C |
| G2 vs. G4 | 13.25 | Yes | ** | 0.0076 | B-D |
| G3 vs. G4 | 0 | No | ns | >0.9999 | C-D |
|  |  |  |  |  |  |
|  |  |  |  |  |  |
| Test details | Mean rank 1 | Mean rank 2 | Mean rank diff. | n1 | n2 |
|  |  |  |  |  |  |
| G1 vs. G2 | 19.1 | 29.8 | -10.7 | 10 | 10 |
| G1 vs. G3 | 19.1 | 16.55 | 2.55 | 10 | 10 |
| G1 vs. G4 | 19.1 | 16.55 | 2.55 | 10 | 10 |
| G2 vs. G3 | 29.8 | 16.55 | 13.25 | 10 | 10 |
| G2 vs. G4 | 29.8 | 16.55 | 13.25 | 10 | 10 |
| G3 vs. G4 | 16.55 | 16.55 | 0 | 10 | 10 |

|  | G1 | G2 | G3 | G4 |
| --- | --- | --- | --- | --- |
| Number of values | 10 | 10 | 10 | 10 |
|  |  |  |  |  |
| Minimum | 0 | 0 | 0 | 0 |
| 25% Percentile | 0 | 0 | 0 | 0 |
| Median | 0 | 10 | 0 | 0 |
| 75% Percentile | 15 | 5759 | 0 | 0 |
| Maximum | 60 | 12354 | 0.0001 | 0.0001 |
|  |  |  |  |  |
| Mean | 12 | 2664 | 1e-005 | 1e-005 |
| Std. Deviation | 25.3 | 4641 | 3.162e-005 | 3.162e-005 |
| Std. Error of Mean | 8 | 1468 | 1e-005 | 1e-005 |
|  |  |  |  |  |
| Lower 95% CI | -6.097 | -655.9 | -1.262e-005 | -1.262e-005 |
| Upper 95% CI | 30.1 | 5984 | 3.262e-005 | 3.262e-005 |
|  |  |  |  |  |
| Mean ranks | 19.1 | 29.8 | 16.55 | 16.55 |

**TORQUE AND DETORQUE CRUDE DATA**

| T | | | | | | | | | | DT | | | | | | | | | |
| --- | --- | --- | --- | --- | --- | --- | --- | --- | --- | --- | --- | --- | --- | --- | --- | --- | --- | --- | --- |
| 20 | 20 | 20 | 20 | 20 | 20 | 20 | 20 | 20 | 20 | 19.6 | 20 | 19.8 | 20 | 20.1 | 20 | 20.3 | 19.8 | 19.9 | 20.1 |
| 20 | 20 | 20 | 20 | 20 | 20 | 20 | 20 | 20 | 20 | 18 | 20 | 20 | 21 | 21 | 19 | 20 | 20 | 20 | 20 |
| 20 | 20 | 20 | 20 | 20 | 20 | 20 | 20 | 20 | 20 | 55 | 56 | 58 | 55 | 58 | 52 | 58 | 52 | 56 | 52 |
| 20 | 20 | 20 | 20 | 20 | 20 | 20 | 20 | 20 | 20 | 17.8 | 19.7 | 19.9 | 20.3 | 20.1 | 18.9 | 19.7 | 19.2 | 19.9 | 19.6 |

**NORMALITY TEST TORQUE AND DETORQUE**

|  | G1 | G2 | G3 | G4 | G1.a | G2.a | G3.a | G4.a |
| --- | --- | --- | --- | --- | --- | --- | --- | --- |
| Number of values | 10 | 10 | 10 | 10 | 10 | 10 | 10 | 10 |
|  |  |  |  |  |  |  |  |  |
| Minimum | 20 | 20 | 20 | 20 | 19.6 | 18 | 52 | 17.8 |
| 25% Percentile | 20 | 20 | 20 | 20 | 19.8 | 19.75 | 52 | 19.13 |
| Median | 20 | 20 | 20 | 20 | 20 | 20 | 55.5 | 19.7 |
| 75% Percentile | 20 | 20 | 20 | 20 | 20.1 | 20.25 | 58 | 19.95 |
| Maximum | 20 | 20 | 20 | 20 | 20.3 | 21 | 58 | 20.3 |
|  |  |  |  |  |  |  |  |  |
| Mean | 20 | 20 | 20 | 20 | 19.96 | 19.9 | 55.2 | 19.51 |
| Std. Deviation | 0 | 0 | 0 | 0 | 0.1955 | 0.8756 | 2.486 | 0.7264 |
| Std. Error of Mean | 0 | 0 | 0 | 0 | 0.06182 | 0.2769 | 0.786 | 0.2297 |
|  |  |  |  |  |  |  |  |  |
| Lower 95% CI of mean | 20 | 20 | 20 | 20 | 19.82 | 19.27 | 53.42 | 18.99 |
| Upper 95% CI of mean | 20 | 20 | 20 | 20 | 20.1 | 20.53 | 56.98 | 20.03 |
|  |  |  |  |  |  |  |  |  |
| Sum | 200 | 200 | 200 | 200 | 199.6 | 199 | 552 | 195.1 |
|  |  |  |  |  |  |  |  |  |
| Shapiro-Wilk normality test |  |  |  |  |  |  |  |  |
| W |  |  |  |  | 0.9674 | 0.8203 | 0.8477 | 0.8558 |
| P value |  |  |  |  | 0.8657 | 0.0256 | 0.0545 | 0.0680 |
| Passed normality test (alpha=0.05)? |  |  |  |  | Yes | No | Yes | Yes |
| P value summary |  |  |  |  | ns | * | ns | ns |
|  |  |  |  |  |  |  |  |  |
| KS normality test |  |  |  |  |  |  |  |  |
| KS distance | 1 | 1 | 1 | 1 | 0.1811 | 0.3455 | 0.201 | 0.2493 |
| P value | <0.0001 | <0.0001 | <0.0001 | <0.0001 | >0.1000 | 0.0013 | >0.1000 | 0.0784 |
| Passed normality test (alpha=0.05)? | No | No | No | No | Yes | No | Yes | Yes |
| P value summary | **** | **** | **** | **** | ns | ** | ns | ns |

**TWO-WAY ANOVA TORQUE AND DETORQUE**

| Table Analyzed | T e DT |  |  |  |  |
| --- | --- | --- | --- | --- | --- |
|  |  |  |  |  |  |
| Two-way RM ANOVA | Matching: Both factors |  |  |  |  |
| Alpha | 0.05 |  |  |  |  |
|  |  |  |  |  |  |
| Source of Variation | % of total variation | P value | P value summary | Significant? |  |
| Row Factor | 42.88 | <0.0001 | **** | Yes |  |
| Column Factor | 13.62 | <0.0001 | **** | Yes |  |
| Interaction: Row Factor x Column Factor | 42.88 | <0.0001 | **** | Yes |  |
| Interaction: Row Factor x Subjects | 0.184 |  |  |  |  |
| Interaction: Column Factor x Subjects | 0.1242 |  |  |  |  |
| Subjects | 0.1242 |  |  |  |  |
|  |  |  |  |  |  |
| ANOVA table | SS | DF | MS | F (DFn, DFd) | P value |
| Row Factor | 4703 | 3 | 1568 | F (3, 27) = 2098 | P<0.0001 |
| Column Factor | 1494 | 1 | 1494 | F (1, 9) = 987.1 | P<0.0001 |
| Interaction: Row Factor x Column Factor | 4703 | 3 | 1568 | F (3, 27) = 2098 | P<0.0001 |
| Interaction: Row Factor x Subjects | 20.18 | 27 | 0.7473 |  |  |
| Interaction: Column Factor x Subjects | 13.62 | 9 | 1.513 |  |  |
| Subjects | 13.62 | 9 | 1.513 |  |  |
| Residual | 20.18 | 27 | 0.7473 |  |  |

| Table Analyzed | T e DT normalidade |
| --- | --- |
|  |  |
| Kruskal-Wallis test |  |
| P value | <0.0001 |
| Exact or approximate P value? | Approximate |
| P value summary | **** |
| Do the medians vary signif. (P < 0.05)? | Yes |
| Number of groups | 8 |
| Kruskal-Wallis statistic | 41.46 |
|  |  |
| Data summary |  |
| Number of treatments (columns) | 8 |
| Number of values (total) | 80 |

| Number of families | 1 |  |  |  |  |
| --- | --- | --- | --- | --- | --- |
| Number of comparisons per family | 28 |  |  |  |  |
| Alpha | 0.05 |  |  |  |  |
|  |  |  |  |  |  |
| Dunn's multiple comparisons test | Mean rank diff. | Significant? | Summary | Adjusted P Value |  |
|  |  |  |  |  |  |
| G1 vs. G2 | 0 | No | ns | >0.9999 | A-B |
| G1 vs. G3 | 0 | No | ns | >0.9999 | A-C |
| G1 vs. G4 | 0 | No | ns | >0.9999 | A-D |
| G1 vs. G1.a | 3.5 | No | ns | >0.9999 | A-E |
| G1 vs. G2.a | 1.1 | No | ns | >0.9999 | A-F |
| G1 vs. G3.a | -36.5 | Yes | ** | 0.0018 | A-G |
| G1 vs. G4.a | 19.9 | No | ns | 0.8147 | A-H |
| G2 vs. G3 | 0 | No | ns | >0.9999 | B-C |
| G2 vs. G4 | 0 | No | ns | >0.9999 | B-D |
| G2 vs. G1.a | 3.5 | No | ns | >0.9999 | B-E |
| G2 vs. G2.a | 1.1 | No | ns | >0.9999 | B-F |
| G2 vs. G3.a | -36.5 | Yes | ** | 0.0018 | B-G |
| G2 vs. G4.a | 19.9 | No | ns | 0.8147 | B-H |
| G3 vs. G4 | 0 | No | ns | >0.9999 | C-D |
| G3 vs. G1.a | 3.5 | No | ns | >0.9999 | C-E |
| G3 vs. G2.a | 1.1 | No | ns | >0.9999 | C-F |
| G3 vs. G3.a | -36.5 | Yes | ** | 0.0018 | C-G |
| G3 vs. G4.a | 19.9 | No | ns | 0.8147 | C-H |
| G4 vs. G1.a | 3.5 | No | ns | >0.9999 | D-E |
| G4 vs. G2.a | 1.1 | No | ns | >0.9999 | D-F |
| G4 vs. G3.a | -36.5 | Yes | ** | 0.0018 | D-G |
| G4 vs. G4.a | 19.9 | No | ns | 0.8147 | D-H |
| G1.a vs. G2.a | -2.4 | No | ns | >0.9999 | E-F |
| G1.a vs. G3.a | -40 | Yes | *** | 0.0003 | E-G |
| G1.a vs. G4.a | 16.4 | No | ns | >0.9999 | E-H |
| G2.a vs. G3.a | -37.6 | Yes | ** | 0.0010 | F-G |
| G2.a vs. G4.a | 18.8 | No | ns | >0.9999 | F-H |
| G3.a vs. G4.a | 56.4 | Yes | **** | <0.0001 | G-H |

|  | G1 | G2 | G3 | G4 | G1.a | G2.a | G3.a | G4.a |
| --- | --- | --- | --- | --- | --- | --- | --- | --- |
| Number of values | 10 | 10 | 10 | 10 | 10 | 10 | 10 | 10 |
|  |  |  |  |  |  |  |  |  |
| Minimum | 20 | 20 | 20 | 20 | 19.6 | 18 | 52 | 17.8 |
| 25% Percentile | 20 | 20 | 20 | 20 | 19.8 | 19.75 | 52 | 19.13 |
| Median | 20 | 20 | 20 | 20 | 20 | 20 | 55.5 | 19.7 |
| 75% Percentile | 20 | 20 | 20 | 20 | 20.1 | 20.25 | 58 | 19.95 |
| Maximum | 20 | 20 | 20 | 20 | 20.3 | 21 | 58 | 20.3 |
|  |  |  |  |  |  |  |  |  |
| Mean | 20 | 20 | 20 | 20 | 19.96 | 19.9 | 55.2 | 19.51 |
| Std. Deviation | 0 | 0 | 0 | 0 | 0.1955 | 0.8756 | 2.486 | 0.7264 |
| Std. Error of Mean | 0 | 0 | 0 | 0 | 0.06182 | 0.2769 | 0.786 | 0.2297 |
|  |  |  |  |  |  |  |  |  |
| Lower 95% CI | 20 | 20 | 20 | 20 | 19.82 | 19.27 | 53.42 | 18.99 |
| Upper 95% CI | 20 | 20 | 20 | 20 | 20.1 | 20.53 | 56.98 | 20.03 |
|  |  |  |  |  |  |  |  |  |
| Mean ranks | 39 | 39 | 39 | 39 | 35.5 | 37.9 | 75.5 | 19.1 |
